# Feature-Based Classification of Amino Acid Substitutions outside Conserved Functional Protein Domains

**DOI:** 10.1155/2013/948617

**Published:** 2013-11-17

**Authors:** Branislava Gemovic, Vladimir Perovic, Sanja Glisic, Nevena Veljkovic

**Affiliations:** Centre for Multidisciplinary Research and Engineering, Vinca Institute of Nuclear Sciences, University of Belgrade, 12-14 Mihajla Petrovica Alasa, 11001 Belgrade, Serbia

## Abstract

There are more than 500 amino acid substitutions in each human genome, and bioinformatics tools irreplaceably contribute to determination of their functional effects. We have developed feature-based algorithm for the detection of mutations outside conserved functional domains (CFDs) and compared its classification efficacy with the most commonly used phylogeny-based tools, PolyPhen-2 and SIFT. The new algorithm is based on the informational spectrum method (ISM), a feature-based technique, and statistical analysis. Our dataset contained neutral polymorphisms and mutations associated with myeloid malignancies from epigenetic regulators ASXL1, DNMT3A, EZH2, and TET2. PolyPhen-2 and SIFT had significantly lower accuracies in predicting the effects of amino acid substitutions outside CFDs than expected, with especially low sensitivity. On the other hand, only ISM algorithm showed statistically significant classification of these sequences. It outperformed PolyPhen-2 and SIFT by 15% and 13%, respectively. These results suggest that feature-based methods, like ISM, are more suitable for the classification of amino acid substitutions outside CFDs than phylogeny-based tools.

## 1. Introduction

Next generation sequencing technologies are revolutionizing genetics through enabling sequencing of whole genomes and exomes and increasing our ability to connect different genotypes to specific phenotypes. With the ending of phase I of the 1000 genomes project, we are facing the fact that human genome has on average around 3.7 million single nucleotide polymorphisms (SNPs) of which 24 000 are in GENCODE regions [1, 2]. More than 500 SNPs per exome affect protein sequence [3, 4], leading to amino acid substitutions (AASs). The major focus is on identification of genetic variants that disrupt molecular functions and cause human diseases. This is a particularly challenging task for complex diseases, like cancers, where each patient, with unique set of alterations, is in need of personalized approach [5].

There are three key *in silico* strategies for prediction of functional effects of AASs (reviewed in, e.g., [6, 7]). The first group of methods approaching this issue from evolutionary perspective relies on the multiple sequence alignments (MSA) of homologous proteins. Methods, such as PANTHER [8], PhD-SNP [9], and SIFT [10], presume that functionally important regions of a protein will be conserved throughout the evolution and assume direct connection between conservation of a residue and the functional effect of the AAS. The second strategy combines scores from MSA with structural information as well as patterns of physicochemical properties of amino acid substitutions. For predictions, these methods use machine learning algorithms, such as random forest—MutPred [11], neural networks—SNAP [12], or Bayesian classification—PolyPhen-2 [13]. The third strategy is MSA-independent sequence analysis relying on the prediction of the effect of an AAS on the sequence structural patterns. These unobvious patterns of physicochemical or biochemical features correlate with protein structure and biological functions ([14] and references herein). In general, the methods that unravel sequence periodicities encompass two steps: first, the sequence represented in alphabetic code is transformed into series of numbers by assigning to each amino acid a value of selected parameter and then these series of numbers are transformed by digital-signal processing techniques such as wavelet and Fourier transformations (FT). PseAAC is one method relying on the analysis of the hydrophobic, hydrophilic, side chain mass, pK and pI patterns for prediction of protein attributes, like subcellular localization and protein structural class [15]. On the other hand, ISM method based on electron ion interaction potential (EIIP) pattern conversion [16] has been successfully applied in functional annotation of AASs [17–20], as well as in the study of protein domains and their associations with disease [21].

The evolutionarily conserved amino acids are preferentially found in CFDs that play the most important roles in the biological function of proteins, such as the active site of enzymes. Tools relying on evolutionary conservation have better applicability in the identification of variants associated with monogenic diseases than with complex diseases, as conservation patterns of variants known to be linked to common complex diseases appear to be indistinguishable from the patterns of polymorphisms occurring in the general population [22]. Of note, according to COSMIC database, more than 50% of AASs associated with cancers were shown to be outside CFDs [23]. We hypothesize here that these AASs might impair sequence patterns which are not necessarily identical with CFDs and, therefore, could be annotated more efficiently with feature-based tool, ISM, compared to two of the most widely used tools the PolyPhen-2 and SIFT, which both account for evolutionarily conserved protein patterns.

As a model set for testing our hypotheses, we chose four epigenetic regulators ASXL1, EZH2, DNMT3A, and TET2, which are frequently mutated in the myeloid malignancies comprising around 25% of all hematological malignancies, with annual incidence of 7.6 per 100 000 [24]. The most common is acute myeloid leukemia (AML), which occurs de novo or evolves from chronic stages that include myelodisplastic syndromes (MDS), myeloproliferative neoplasms (MPN), and MDS/MPN combined disorders. Mutations in epigenetic regulators lead to anomalies in epigenetic profiles, which is a hallmark of myeloid malignancies and frequent molecular marker of worse prognosis [25–32]. DNMT3A and TET2 are enzymes constituting DNA methylation/demethylation machinery [33, 34], while both EZH2 and ASXL1 achieve their functions through the methylation of histones [35, 36]. Importantly, it has been widely assumed that these molecules actively contribute to the transformation of chronic to acute stages, which suggest their employment as clinical biomarkers (reviewed in [37]).

Aiming to investigate the predictive power of alignment-free approach, ISM, we develop a method to differentiate between neutral versus pathogenic AASs. The presented results point to the limitations of MSA-based tools, PolyPhen-2, and SIFT, to detect mutations that are not part of CFDs and showed that feature-based ISM tool performs much better on this task.

## 2. Materials and Methods

### 2.1. Sequences and Polymorphisms

Wild type sequences of ASXL1, EZH2, DNMT3A, and TET2 were retrieved from UniProtKB database [40]. Since we were interested in analysis of polymorphisms outside CFDs (non-CFDs regions—nCFDs), they were identified in the relevant literature (Table 1).

Mutations were collected from the literature, through the screening of PubMed knowledgebase and from COSMIC database [41]. To label an AAS as a mutation, besides its association with a myeloid malignancy, we looked in original papers for evidence of its somatic nature. SNPs were collected from the literature and dbSNP database. There were two criteria to label an AAS as an SNP: the first included evidence in original papers of its presence in germline, and the second implied described frequency of the polymorphism in healthy population.

### 2.2. SIFT and PolyPhen-2

SIFT uses sequence homology to predict the effect of an AAS on protein function, considering the position at which the substitution occurred and the type of amino acid change. In the first step, SIFT creates MSA containing the sequences, related to the given protein sequence and, then, it calculates the probability that the amino acid change is tolerated. In this study, we had to transform SIFT scores so they could be compared with other tools, and we calculated SIFT score = 1 − SIFT score_(org)_, where SIFT score_(org)_ is the score originally retrieved from the SIFT tool. For example, SIFT score_(org)_ of 0.01 associated with a mutation and 0.88 that of with an associated SNP were this way transformed into 0.99 and 0.12, making the higher score related to mutation and lower to SNP. We used single protein tool SIFT sequence, with default values of median conservation of sequences (3.0). The PSI-BLAST search was applied on UniRef90 database, and sequences with the similarity level of 90% or more to the query sequence were removed from the alignment. Binary classification was done by annotating AAS with SIFT score_(org)_ <0.05 as mutation and AAS with SIFT score_(org)_ >0.05 as SNP.

PolyPhen-2 bases its predictions of damaging effects of missense mutations on eight sequence-based and three structure-based features, which were selected using machine learning. The functional effect of an amino acid substitution is predicted based on the calculated Naïve Bayes probabilistic score. A mutation is classified as probably damaging when the score is above 0.85, possibly damaging when the score is above 0.15, and the remaining as benign. For the binary classification, we adopted cutoff for probabilistic score of 0.5, so substitutions with the score above this cutoff were considered to be mutations and those below the cutoff to be SNPs. We used default values for query options and HumDiv-trained version of PolyPhen-2, as this is recommended for the evaluation of mutations involved in complex phenotypes.

### 2.3. ISM Algorithm

**Figure 1:**
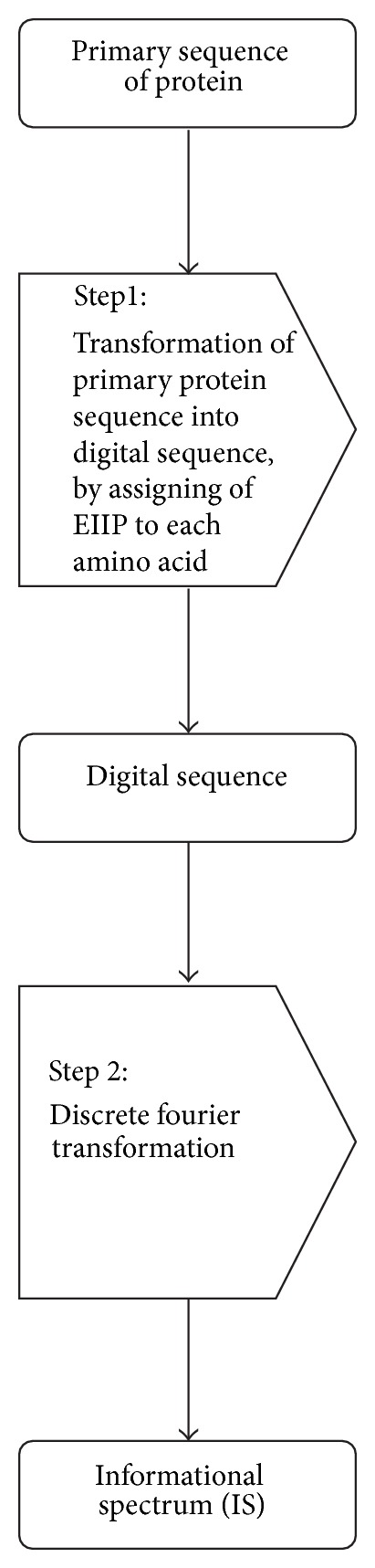
Scheme for the ISM procedure.

ISM uses FT as a mathematical tool to highlight the periodical structural patterns in the protein sequences and assesses the effect of each AAS on sequence and consequently on the correlating biological function of the protein. Procedure, schematically presented in Figure 1, comprises two steps. The first step includes transformation of amino acid sequence into sequence of numbers by assigning an EIIP value to a matching amino acid (Table 2). EIIP values approximate energy of valence electrons and were calculated for each amino acid using the general model pseudopotential as follows [42]:

(1) W=0.25  Z∗sin⁡(1.04πZ∗)2π.

Z*, that represents the average quasivalence number, is calculated as

(2) Z∗=1N∑i=1mniZi,

where *Z*_*i*_ is the valence number of the *i*th atomic component, *n*_*i*_ is the number of atoms of the *i*th component, *m* is the number of atomic components in the molecule, and *N* is the total number of atoms. It was previously shown that the periodicity of EIIP distribution along the protein sequence correlates with biological activity of a protein, especially with its specific interactions with ligands and other proteins (reviewed in [16]).

The second step is the conversion of this sequence of numbers using FT, which is defined as

(3) X(n)=∑m=1Nx(m)e−i2πn(m−1)/N, n=1,2,…,N2,

where *x*(*m*) is the *m*th member of a given numerical series, *N* is the total number of points in the series, and *X*(*n*) are discrete FT coefficients. FT approximates a string of numerical values representing a protein sequence by a linear combination of trigonometric functions with different periodicities, and FT coefficients describe the amplitude, phase, and frequency of these sinusoids (periodical functions) from the original signal. Relevant information for protein analysis is extracted into informational spectrum (IS), an energy density spectrum defined as

(4) S(n)=X(n)X∗(n)=|X(n)|2, n=1,2,…,N2,

where *X*(*n*) are discrete FT coefficients and X*(*n*) are complex conjugate discrete FT coefficients. This way sequences are transformed into discrete signals, where the points in numerical series are assumed to be equidistant (distance is arbitrary set to *d* = 1). The maximum frequency in the spectrum is then 1/2*d* = 0.5. 

Peaks in the IS correspond to the functions with certain periodicities that contribute to the original signal greater than functions with other periodicities. So, IS can be used to detect latent sequence periodicities at a certain frequency and, with the assumption that characteristics of sequence repeats uniquely identify structural repeats, IS can recognize difficult structural patterns in the protein sequences [43, 44]. Thus, the information primary represented as amino acid sequence is, through described two steps, transformed into IS, where peaks correspond to structural patterns and consequently specific biological functions of analyzed protein. 

ISM was the basis for the algorithm for functional annotation of AASs, developed in this study. Statistical significance of ISM frequencies was assessed with Mann-Whitney *U* Test, with significance level of *p* < 0.05. The algorithm comprises five steps as follows.

(1) Creation of ISs for wild type sequences and all sequences with substituted amino acids. Wild type IS is a reference spectrum and it will be used in step (5) to determine cutoffs, while ISs of sequences with AASs were scored in step (2).
(2) ISM scoring system: scores are calculated as deviations of amplitude values of sequence with AAS from the matching values of wild type sequence, for each frequency in the IS as follows:
  (5) S(i,j)=A(fj)vari−A(fj)wt,  i=1,2,…,N,  j=1,2,…,M,
  where *N* is the number of AASs and *M* is the number of frequencies in the IS. These ISM scores are the basis for statistical analysis.
(3) Use of Mann-Whitney *U* Test for the frequency with highest value of amplitude in the IS of wild type sequence in order to detect significance of this frequency in classification of AASs into deleterious mutations and neutral SNPs. If this did not show to be statistically significant, the same analysis was done for other frequencies in descending order of their values of amplitudes.
(4) The first frequency that shows statistical significance in discriminating sequences with mutations and SNPs is chosen as a classifier.
(5) The value of amplitude for selected frequency in the IS of wild type sequence is used as a cutoff separating sequences with mutations from those with SNPs.

ISM algorithm must be applied on each protein separately, which means that significant frequencies and cutoffs are different for different proteins. Also, it is impossible to determine beforehand if a mutation increases or decreases the amplitude on the significant frequency compared to the wild type, so this can be concluded only after all the five steps of the algorithm are performed.

## 3. Statistics

The efficacy of prediction tools were assessed by the number of true positives (TP), true negatives (TN), false positives (FP), and false negatives (FN). The parameters for evaluation were as follows:

 accuracy = TP + TN/TP + TN + FP + FN,
 precision = TP/TP + FP,
 negative predictive value (NPV) = TN/TN + FN,
 sensitivity = TP/TP + FN,
 specificity = TN/TN + FP.

Crosstabulation was done for categorical variables and, Fisher's exact test was used for the assessment of their statistical significance.

We also constructed receiver operating characteristic (ROC) curves for SIFT, PolyPhen-2, and ISM scores and used area under the curve (AUC) to evaluate predictions of these different methods.

## 4. Results

### 4.1. Polymorphisms in Epigenetic Regulators ASXL1, EZH2, DNMT3A, and TET2

Our dataset is summarized in Table 3 and shown in detail in Supplementary Material available online at http://dx.doi.org/10.1155/2013/948617. It contains 314 AASs in epigenetic regulators ASXL1, EZH2, DNMT3A, and TET2. 194 disease-associated and somatically acquired polymorphisms are labeled as mutations, while 120 germline or polymorphisms present in healthy population are labeled as SNPs. The most frequent mutations in the dataset are from AML cases (45%), and 12%, 13%, and 7% of mutations are from MDS, MPN, and MDS/MPN, respectively. The rest of the mutations were detected in two or more different myeloid malignancies.

A subset of AASs in nCFDs contains 159 polymorphisms, 108 SNPs and 51 mutations (Table 3). Mutations from AML make 41% of this subset, while 10%, 27%, and 14% of mutations are from MDS, MPN and MDS/MPN, respectively. Only 8% of mutations were reported in two or more myeloid malignancies.

### 4.2. Performances of PolyPhen-2 and SIFT

**Figure 2:**
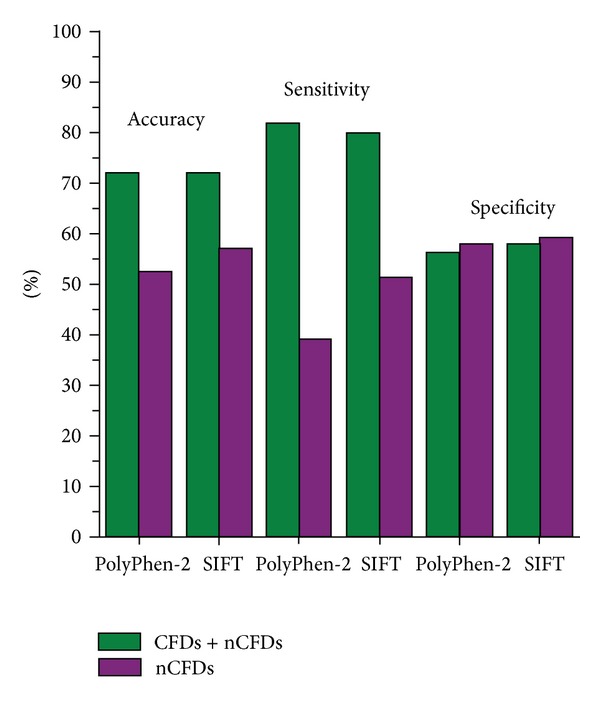
Performance of PolyPhen-2 and SIFT on the entire dataset (CFDs and nCFDs) and on the subset of variations outside CFDs (nCFDs).

When we evaluated performance of PolyPhen-2 and SIFT on our entire dataset of 314 AASs, both tools had overall accuracy of 72%, with considerably higher values of sensitivity compared to specificity (Figure 2). The same analysis of the subset of 159 AASs positioned in nCFDs showed decrease in overall accuracy, reaching values of 52% and 57% for PolyPhen-2 and SIFT, respectively (Figure 2). The specificity remained the same, independently of the position of the AASs. However, the value of sensitivity dropped largely when compared entire dataset and the subset, from 82% to 39% for PolyPhen-2 and from 80% to 51% for SIFT. This comes from high number of false negative predictions of AASs outside CFDs. 

### 4.3. Predictions Based on the ISM Algorithm

We applied ISM algorithm to identify classifier frequencies for discrimination between group of sequences with mutations and group of sequences with SNPs in ASXL1, EZH2, DNMT3A, and TET2.

**Figure 3:**
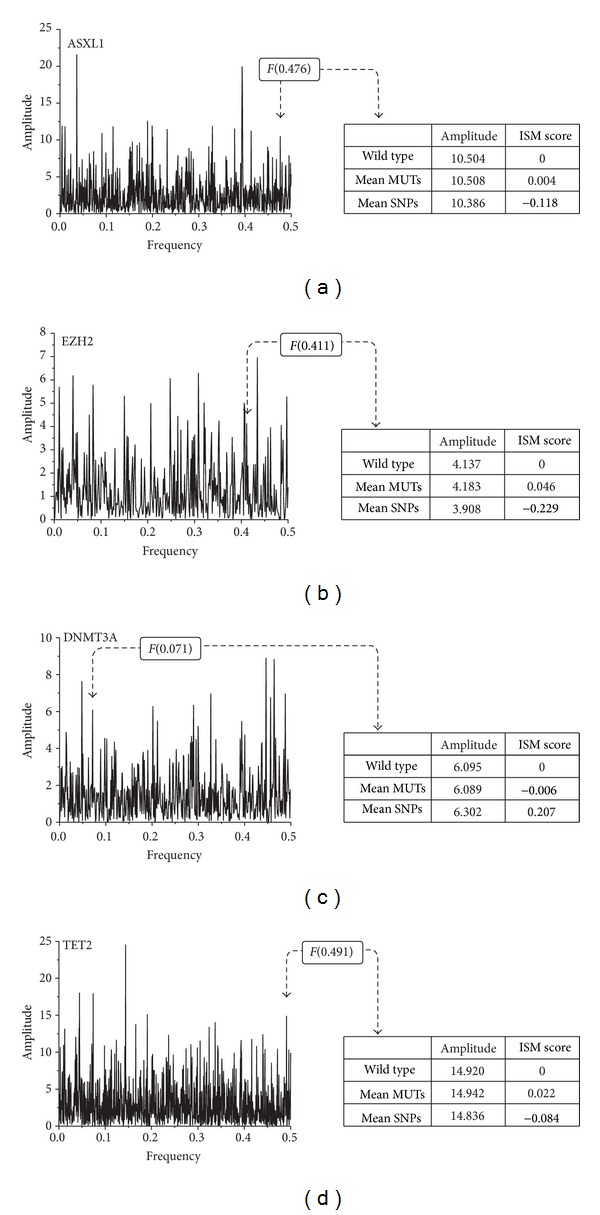
Process for the selection of significant frequencies from the spectra of ASXL1 (a), EZH2 (b), DNMT3A (c), and TET2 (d).

**Figure 4:**
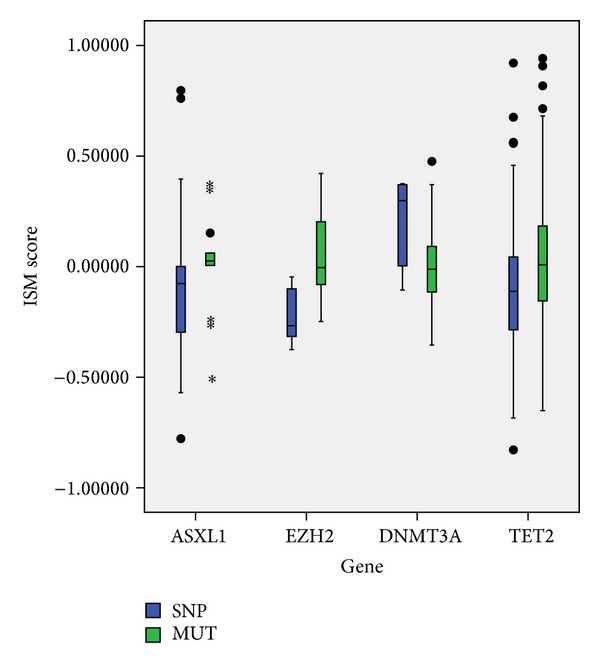
Distribution of ISM scores.

Our first step encompassed creation of ISs for wild type sequence of ASXL1 and 76 sequences with AASs. Second, we calculated ISM scores for each frequency in the IS. In the third step, we performed Mann-Whitney *U* Test on these scores related to the frequency with highest amplitude value in IS of wild type sequence—*F*(0.036). As it did not significantly discriminates between SNPs and mutations, we applied the same statistical test for the next highest peak frequency in the spectrum. We went on with this procedure until we identified IS peak frequency *F*(0.476) that discriminate disease related mutations (*p* = 0.018) (Figure 3(a)). 75% of sequences with SNPs had lower and 77% of sequences with mutations had higher values of amplitudes compared to wild type (Figure 4).

EZH2 is frequently mutated in lymphoid malignancies, with the hot spot on Tyr641 [45]; however, mutations in myeloid malignancies are spread throughout the entire sequence with no hot spot. ISM algorithm identified frequency *F*(0.411) that significantly discriminates sequences with SNPs and mutations, with *p* = 0.003 (Figure 3(b)). Six SNPs containing sequences had amplitude value corresponding to this frequency below the value of wild type, while approximately half of sequences with mutations had higher values of amplitudes than wild type (Figure 4).

In DNMT3A sequence, 6 SNPs and 41 mutations were separated at IS frequency *F*(0.071) with *p* = 0.041 (Figure 3(c)). Contrary to the ASXL1 and EZH2,the majority of sequences with SNPs had amplitude values above wild type value (83%), while more than half of the sequences with mutations (51%) had corresponding amplitudes lower than wild type (Figure 4).

Finally, we analyzed 45 TET2 sequences with SNPs and 121 with mutations. IS frequency *F*(0.491) was shown to be significant classifier (*p* = 0.025) (Figure 3(d)) separating sequences with SNPs (60% below wild type value) and with mutations (55% above wild type value) (Figure 4). Since TET2 variations make the largest proportion of our dataset, we used them for cross-validation of our method for frequency selection. We randomly split them into five groups, and each time we submitted four different groups to the ISM-based algorithm. All analyses resulted in the identification of *F*(0.491) as the most important frequency, which indicates minimal bias in our performance evaluation.

### 4.4. Performance of ISM Algorithm on AASs outside CFDs and Comparison with PolyPhen-2 and SIFT

This research is focused on predictions of functional effects of AASs in nCFDs. We compared predictive power of ISM algorithm and commonly used MSA-based PolyPhen-2 and SIFT on the subset of our data, which contained 108 SNPs and 51 mutations.

ISM scores represent the difference between mutated and wild type sequence. Higher ISM scores were associated with mutations in ASXL1, EZH2, and TET2, but in DNMT3A this relation was inversely proportional. In order to allow ISM scores for all analyzed genes to be drawn to a same scale and compared, we transformed DNMT3A by multiplication with factor *a* = −1. In this way, the DNMT3A scores, 0.37473 associated with an SNP and −0.24349 associated with a mutation, were transformed into SNP related −0.37473 and mutation related 0.24349. 

**Figure 5:**
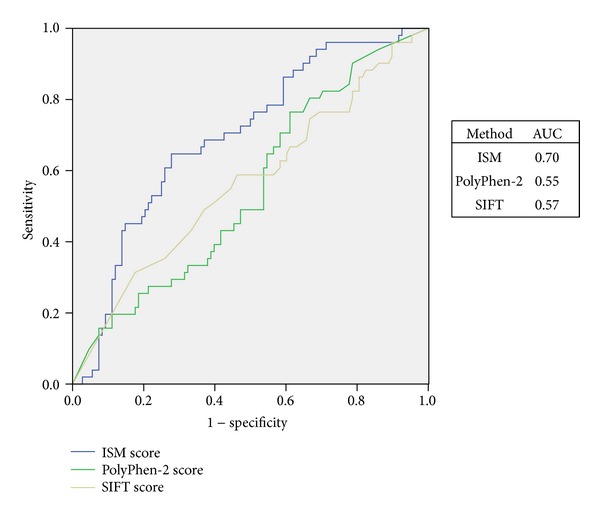
ROC curves on the ISM, PolyPhen-2 and SIFT scores for nCFD variations.

**Figure 6:**
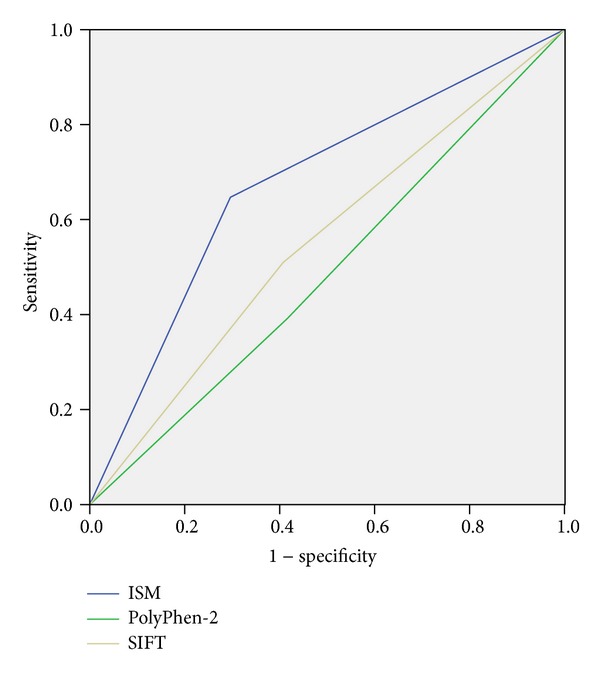
ROC curves for binary classification.

Further, we created ROC curves and found that ISM algorithm outperformed PolyPhen-2 and SIFT, with the AUC values 0.70, 0.55, and 0.57, respectively (Figure 5). In addition, we evaluated binary classification. Accuracy of ISM for this dataset was 17% and 12% better than that of PolyPhen-2 and SIFT, respectively (Table 4). The overall better performance of ISM is also shown through 17% and 13% higher values of AUC compared to PolyPhen-2 and SIFT, respectively (Figure 6). It is important to stress out that sensitivity measuring false negative rate shows better performance of ISM algorithm compared to PolyPhen-2 and SIFT for 26% and 14%, respectively. Finally, cross tabulation and Fisher's exact test showed that only ISM-based classification of AASs in nCFDs is statistically significant, with *p* < 0.001 (Table 4).

## 5. Discussion

Most computational methods that predict deleterious AASs are sequence- or structure-based and presume that most disease-causing AASs affects evolutionarily conserved domains. PolyPhen-2 and SIFT recognize AASs clustered in CFDs with high accuracy, assuming that residue in the conserved position affect protein function. In our dataset, 97.2% and 90.2% CFD mutations were predicted as damaging by PolyPhen-2 and SIFT, respectively. This 7% difference is perhaps due to the sequence-based feature of PolyPhen-2, named pfam_hit, that accounts for position of the mutation within/outside a protein domain as defined by Pfam, which is a database of known CFDs [46]. However, compared to overall performances of PolyPhen-2 and SIFT evaluated on HumDiv and HumVar datasets, their accuracy and specificity on our dataset are lower, which is in accordance with previous study of four other cancer genes, BRCA1, MSH2, MLH1, and TP53 [47]. For AASs outside conserved regions, this low specificity is accompanied with the significantly decreased value of sensitivity, as well. Weak performance of PolyPhen-2 and SIFT on the subset of AASs positioned in nCFDs suggests that conservation of amino acid position in these parts of proteins does not account for its functional role. Predictions based on homology and evolutionary conservation often cannot describe the underlying mechanisms of how substitutions result in changes in the protein phenotypes. In that regard, ISM is useful as it sheds light on the effect that given AAS has on protein-protein interactions. This technique allows the detection or definition of amplitude/frequency pairs determining the specific long-range recognition between interacting proteins [16, 48]. Therefore, the disruption of EIIP profile along a protein, which is manifested in ASXL1, EZH2, and TET2 through the increase of amplitudes on *F*(0.476), *F*(0.411), and *F*(0.491), respectively, and in DNMT3A through the decrease in amplitude on *F*(0.071), is probably associated with the significant effects on large interaction networks. This is supported by the observation that cancer proteins are characterized by the promiscuity in transient protein-protein interactions [49] which frequently engage not conserved residues [50].

In future, it will be important to consider IS classification criteria based on more than one IS frequency and therefore accounting for more than one cellular function. This will improve annotation of genes, such as EZH2 in which 3 mutations outside CFDs were correctly classified (L149Q, A384T, and T568I), while three others were incorrectly annotated as SNPs (M134K, C534R and L575P). Detail examinations have shown that correctly classified mutations are from cases with MPN and false negatives are from MDS. This finding implies that IS frequency *F*(0.411) correlates with dysfunction in proliferation that leads to MPN and not differentiation, which is underlying dysfunction of MDS [51].

Besides the effects on functions, some mutations play their pathological roles through affecting the stability of proteins [52]. Actually, it was shown that 75% of mutations in inherited diseases affect protein stability [53]. Recently, metatools have been proposed [54, 55] that appear to achieve better performance by combining prediction scores from multiple tools. In that regard, it would be interesting to combine methods predicting AAS effects on protein stability, such as FoldX [56], CUPSAT [57], or Eris [58], and feature-based methods.

## 6. Conclusions

This work suggests that classical phylogeny-based methods are not suitable for prediction of functional effects of AASs outside CFDs and that these predictions need additional approach. Here, we propose the use of disruption of distribution of EIIP, a physicochemical feature of amino acids, estimated by the FT-based ISM technique, as a suitable approach to detect mutations outside CFDs. We see no obstacles to apply this approach for the prediction of functional effects of AASs outside CFDs on any other type of proteins, hoping that this will bring us one step closer to understanding mutations as molecular markers of diseases.

## Supplementary Material

The amino acid substitutions in ASXL1, EZH2, DNMT3 and TET2 were collected from three sources: literature, COSMIC database and dbSNP database. Literature was scanned for the information on the substitutions in these four genes and their somatic/germline origin. The search of these sources resulted in the dataset of 314 amino acid substitutions. 194 mutations met the two following criteria: i) they were shown to be somatic in the literature and/or ii) they were present in the COSMIC database. The database bellow contains additional information about the type of cancer/s mutations are associated with. For the 120 SNPs, the criteria were as follows: i) they were shown to be in the germline in the literature and/or ii) they were present in the dbSNP database, with known frequency in the general population.

## Figures and Tables

**Table 1:** Sequences, their UniProt IDs, CFDs, and the relevant literature.

Protein	UniProt ID	CFD	Position	Reference
ASXL1	Q8IXJ9	HARE	11–83	
		ASXH	241–369	[32]
		PHD	1506–1541	

EZH2	Q15910	SANT1	159–250	
		SANT2	433–481	[38]
		SET	617–738	

DNMT3A	Q9Y6K1	PWWP	290–348	
		PHD	536–589	[38]
		MTase	638–908	

TET2	Q6N021	BOX1	1104–1478	[39]
		BOX2	1845–2002	

**Table 2:** Abbreviations and EIIP values for amino acids.

Amino acid	Letter code	Numerical code EIIP (Ry)
Leucine	L	0.0000
Isoleucine	I	0.0000
Asparagine	N	0.0036
Glycine	G	0.0050
Valine	V	0.0057
Glutamic acid	E	0.0058
Proline	P	0.0198
Histidine	H	0.0242
Lysine	K	0.0371
Alanine	A	0.0373
Tyrosine	Y	0.0516
Tryptophan	W	0.0548
Glutamine	Q	0.0761
Methionine	M	0.0823
Serine	S	0.0829
Cysteine	C	0.0829
Threonine	T	0.0941
Phenylalanine	F	0.0954
Arginine	R	0.0956
Aspartic acid	D	0.1263

**Table 3:** Number of SNPs and mutations (MUTs) in the dataset.

Gene	SNPs (*n* = 120)		MUTs (*n* = 194)	
	nCFDs	CFDs	nCFDs	CFDs
ASXL1 (*n* = 76)	59	4	12	1
EZH2 (*n* = 25)	4	2	6	13
DNMT3A (*n* = 47)	3	3	6	35
TET2 (*n* = 166)	42	3	27	94

Total	108	12	51	143

**Table 4:** Performance statistics of PolyPhen-2, SIFT, and ISM binary classification of AASs outside CFDs.

	Accuracy	Precision	Sensitivity	Specificity	NPV	AUC
PolyPhen-2 (*p* = 0.863)	0.52	0.31	0.39	0.58	0.67	0.49
SIFT (*p* = 0.236)	0.57	0.37	0.51	0.59	0.72	0.55
ISM (*p* < 0.001)	0.69	0.51	0.65	0.70	0.81	0.68
