# Supplementary material for: Feature-Based Classification of Amino Acid Substitutions outside Conserved Functional Protein Domains
Source: ScientificWorldJournal. 2013 Nov 17;2013:948617. doi: 10.1155/2013/948617 (PMC3855963; doi:10.1155/2013/948617)
Supplement: Supplementary file 1 — The amino acid substitutions in ASXL1, EZH2, DNMT3 and TET2 were collected from three sources: literature, COSMIC database and dbSNP database. Literature was scanned for the information on the substitutions in these four genes and their somatic/germline origin. The search of these sources resulted in the dataset of 314 amino acid substitutions. 194 mutations met the two following criteria: i) they were shown to be somatic in the literature and/or ii) they were present in the COSMIC database. The database bellow contains additional information about the type of cancer/s mutations are associated with. For the 120 SNPs, the criteria were as follows: i) they were shown to be in the germline in the literature and/or ii) they were present in the dbSNP database, with known frequency in the general population. [file 948617.f1.pdf]

Supplement: Detailed dataset – all amino acid substitutions in ASXL1, EZH2, DNMT3A and TET2, with original literature

Gene	AAS	SNP/MUT	Disease	Reference
ASXL1	S25L	SNP		[1]
ASXL1	M29V	SNP		[1]
ASXL1	Q89R	SNP		[2]
ASXL1	A95V	SNP		[1]
ASXL1	C111Y	SNP		[1]
ASXL1	M168L	SNP		[2]
ASXL1	I268F	SNP		[2]
ASXL1	R394H	SNP		[3]
ASXL1	R402Q	MUT	MDS	[3]
ASXL1	S412F	SNP		[2]
ASXL1	E477Q	SNP		[2]
ASXL1	R489G	SNP		[2, 3]
ASXL1	I507N	SNP		[2]
ASXL1	R541W	SNP		[1]
ASXL1	I552V	SNP		[2]
ASXL1	A636V	SNP		[4]
ASXL1	A637T	SNP		[4]
ASXL1	G643V	SNP		[2]
ASXL1	G652S	SNP		[2, 4]
ASXL1	C687R	SNP		[2]
ASXL1	T688M	SNP		[4]
ASXL1	R693Q	SNP		[4]
ASXL1	G704R	SNP		[4]
ASXL1	P722R	SNP		[4]
ASXL1	D741V	SNP		[2]
ASXL1	G744R	MUT	MPN or MDS/MPN	[1]
ASXL1	V751I	SNP		[2]
ASXL1	P779L	SNP		[2]
ASXL1	E788G	MUT	MPN or MDS/MPN	[1]
ASXL1	D799Y	SNP		[2, 4]
ASXL1	T806A	SNP		[1]
ASXL1	P808H	MUT	MPN or MDS/MPN	[1]
ASXL1	T822I	MUT	MPN or MDS/MPN	[1]
ASXL1	L823S	SNP		[3]
ASXL1	K838R	SNP		[1, 2]
ASXL1	S846N	MUT	MDS, MDS/MPN	[5, 6]
ASXL1	E865K	SNP		[2, 4]
ASXL1	M875V	SNP		[4]
ASXL1	D979G	SNP		[1]
ASXL1	L983R	SNP		[2]
ASXL1	T1010M	SNP		[2]
ASXL1	G1026E	SNP		[2]

ASXL1	E1033V	SNP		[7]
ASXL1	P1035S	MUT	MPN or MDS/MPN	[1]
ASXL1	V1046M	SNP		[4]
ASXL1	R1051C	SNP		[4]
ASXL1	P1089S	MUT	MPN or MDS/MPN	[1]
ASXL1	V1092M	MUT	MPN or MDS/MPN	[1]
ASXL1	D1127N	SNP		[2]
ASXL1	M1161V	SNP		[1]
ASXL1	S1166R	SNP		[2, 3]
ASXL1	R1171S	SNP		[8]
ASXL1	G1187V	SNP		[7]
ASXL1	P1197S	SNP		[4]
ASXL1	R1247C	SNP		[2]
ASXL1	M1249V	SNP		[1, 2, 4]
ASXL1	D1252G	SNP		[2]
ASXL1	P1259L	SNP		[2]
ASXL1	V1297I	SNP		[2]
ASXL1	A1312V	SNP		[2-4]
ASXL1	R1316H	SNP		[4]
ASXL1	L1325F	SNP		[2, 9]
ASXL1	G1339E	MUT	MPN, Breast cancer	[10, 11]
ASXL1	A1380V	SNP		[1]
ASXL1	L1395V	SNP		[2, 12]
ASXL1	G1397S	SNP		[2-4]
ASXL1	M1402L	SNP		[1]
ASXL1	S1428P	SNP		[2, 4]
ASXL1	F1463L	SNP		[4]
ASXL1	A1486T	MUT	MDS	[3]
ASXL1	T1494I	SNP		[1]
ASXL1	S1496N	SNP		[1]
ASXL1	S1497G	SNP		[1]
ASXL1	T1498M	MUT	MPN or MDS/MPN	[1]
ASXL1	I1514L	SNP		[2]
ASXL1	C1519Y	MUT	MPN or MDS/MPN	[1]
EZH2	I55M	SNP		[2]
EZH2	M134K	MUT	MDS	[3]
EZH2	L149Q	MUT	MPN	[11, 13]
EZH2	D185H	SNP		[2]
EZH2	E249K	MUT	MDS	[11, 14, 15]
EZH2	Q250E	SNP		[2]
EZH2	N317S	SNP		[2]
EZH2	N361S	SNP		[3]
EZH2	A384T	MUT	MPN, Lung cancer	[11, 14]
EZH2	A482T	SNP		[2, 16]
EZH2	C534R	MUT	MDS	[3]
EZH2	T568I	MUT	MPN	[11, 13]

EZH2	L575P	MUT	MDS	[3]
EZH2	Y641C	MUT	AML, MDS, Lymphoid cancer	[11, 17-19]
EZH2	Q648E	MUT	MDS	[3]
EZH2	A651V	MUT	MDS	[11, 14]
EZH2	D652H	MUT	MDS	[3, 20]
EZH2	D659A	MUT	AML	[11]
EZH2	F655L	MUT	MDS	[3]
EZH2	L669V	MUT	MDS	[11, 15]
EZH2	D672H	MUT	MPN	[11, 13]
EZH2	V674M	MUT	MPN, AML	[11, 13]
EZH2	R679C	MUT	MPN, MDS/MPN	[11, 14]
EZH2	R685H	MUT	MDS, MPN, MDS/MPN, AML	[3, 5, 11, 13, 14, 21, 22]
EZH2	N688T	MUT	AML	[11, 22]
DNMT3A	E30A	SNP		[2]
DNMT3A	G278S	SNP		[2]
DNMT3A	S352N	MUT	AML	[11, 23]
DNMT3A	K468R	MUT	AML	[24]
DNMT3A	V480G	SNP		[2]
DNMT3A	N501S	SNP		[2]
DNMT3A	Y533C	MUT	AML, Lung cancer	[11]
DNMT3A	G543C	MUT	AML, MDS, Lymphoid cancer	[11, 25-28]
DNMT3A	W581C	MUT	AML	[11, 23]
DNMT3A	R635W	MUT	MDS, AML	[11, 27, 29, 30]
DNMT3A	V636M	MUT	AML	[11, 23, 30]
DNMT3A	I655A	MUT	AML	[11, 23]
DNMT3A	R676W	MUT	AML	[11]
DNMT3A	G699S	MUT	MDS	[11]
DNMT3A	S714C	MUT	MDS, AML, Breast cancer	[11, 26, 27, 29-31]
DNMT3A	P718L	MUT	AML	[11, 24]
DNMT3A	R729Q	MUT	AML	[11, 24]
DNMT3A	R729W	MUT	AML	[11, 24]
DNMT3A	F731C	MUT	AML	[11, 23]
DNMT3A	Y735C	MUT	AML	[11, 23]
DNMT3A	R736H	MUT	AML, Lymphoid cancer	[11, 24, 28]
DNMT3A	L737R	MUT	AML	[11, 29]
DNMT3A	A741V	MUT	AML	[11, 24]
DNMT3A	R749C	MUT	MPN, AML	[8, 11]
DNMT3A	R749P	SNP		[2]
DNMT3A	F752L	MUT	AML	[11, 23]
DNMT3A	W753G	MUT	AML	[11, 23]
DNMT3A	L754R	MUT	AML	[11, 23]
DNMT3A	S770W	MUT	AML	[11, 29]
DNMT3A	R771L	MUT	MDS	[11, 29]

DNMT3A	R771Q	MUT	MPN, AML, Lung cancer	[11]
DNMT3A	S775P	SNP		[2]
DNMT3A	R792H	MUT	AML, Lymphoid, Lung cancer	[11, 24, 28]
DNMT3A	W795C	MUT	AML	[11, 23]
DNMT3A	R803S	MUT	AML	[11, 24]
DNMT3A	K829R	MUT	AML	[11, 24]
DNMT3A	K841Q	MUT	AML	[11, 24]
DNMT3A	P849R	MUT	AML	[11, 23]
DNMT3A	M880V	MUT	MPN	[11, 32]
DNMT3A	R882C	MUT	MDS, MPN, MDS/MPN, AML, Lymphoid cancer	[5, 11, 23-28, 30, 32-41]
DNMT3A	R882H	MUT	MDS, MPN, MDS/MPN, AML, Lymphoid cancer	[5, 11, 23-30, 33-49]
DNMT3A	R882P	MUT	MDS/MPN, AML	[5, 11, 24, 26, 27, 36, 37]
DNMT3A	L889P	MUT	AML	[11, 23]
DNMT3A	V897D	MUT	AML	[11, 25, 26]
DNMT3A	P904L	MUT	AML, Lymphoid cancer	[11, 24, 28, 29]
DNMT3A	F909C	MUT	AML	[11, 24]
DNMT3A	A910V	MUT	AML, Endometrial, Intestinal cancer	[11, 23]
TET2	H8D	SNP		[2]
TET2	P22L	MUT	AML	[11, 43]
TET2	P29R	SNP		[2, 9, 50-53]
TET2	L34F	SNP		[2, 9, 50-55]
TET2	P101H	MUT	AML	[11, 51]
TET2	P174H	SNP		[2, 53, 54]
TET2	A215V	SNP		[56]
TET2	V218M	SNP		[2, 9, 50-53, 55]
TET2	A241V	SNP		[57]
TET2	S282F	MUT	AML	[11, 51]
TET2	A308T	MUT	AML	[58]
TET2	N312S	MUT	AML	[11, 54]
TET2	L346P	MUT	MDS/MPN	[11]
TET2	E350K	MUT	AML	[59]
TET2	G355D	SNP		[2, 9, 50-53]
TET2	P363L	SNP		[2, 9, 50-53, 55]
TET2	S393C	SNP		[52]
TET2	P399L	MUT	MDS/MPN	[58]
TET2	G429R	SNP		[2]
TET2	S460F	MUT	MDS/MPN, AML	[52, 54]
TET2	P472A	SNP		[2]
TET2	P474L	SNP		[2]
TET2	P480T	SNP		[56]
TET2	T492S	MUT	AML	[11, 51]
TET2	Q548L	MUT	AML	[11, 51]

TET2	D666G	MUT	MDS/MPN	[54]
TET2	S716L	SNP		[56]
TET2	P761L	SNP		[2]
TET2	N767D	SNP		[2, 60]
TET2	D768V	SNP		[56]
TET2	G773V	MUT	AML	[11, 51]
TET2	E788L	MUT	AML	[11, 61]
TET2	Q810R	SNP		[2, 9]
TET2	R814C	SNP		[62]
TET2	S817T	MUT	MDS/MPN	[58]
TET2	S826I	MUT	AML	[11, 61]
TET2	F868L	SNP		[62]
TET2	A912G	SNP		[2]
TET2	H924R	SNP		[2, 50, 51]
TET2	P941S	MUT	MDS/MPN	[54]
TET2	P968R	SNP		[52]
TET2	E1010D	SNP		[60]
TET2	S1015N	SNP		[56]
TET2	I1025T	SNP		[56]
TET2	S1039L	SNP		[62]
TET2	E1073V	SNP		[54]
TET2	Q1084P	SNP		[2, 50, 51, 53, 54, 56, 58]
TET2	L1120M	SNP		[2]
TET2	C1135Y	MUT	MDS, MPN, MDS/MPN	[54, 63, 64]
TET2	E1144A	MUT	MPN	[11]
TET2	R1167T	MUT	AML	[58]
TET2	C1193W	MUT	MDS, MDS/MPN	[54, 56, 65]
TET2	K1197R	MUT	MDS	[11, 66]
TET2	V1199I	MUT	AML	[11, 51]
TET2	S1204C	MUT	MPN	[54]
TET2	L1210P	MUT	MPN	[67]
TET2	C1211Y	MUT	MDS/MPN, AML	[11, 60, 68, 69]
TET2	R1214W	MUT	MDS, MDS/MPN, AML	[50, 53, 54, 65, 70]
TET2	H1219Q	MUT	AML	[11, 60]
TET2	H1219R	MUT	AML	[11, 60]
TET2	E1222G	MUT	AML	[11, 71]
TET2	L1229R	MUT	AML	[11, 53]
TET2	E1234G	MUT	AML	[11, 71]
TET2	D1242V	MUT	MPN, AML	[11, 54, 72-74]
TET2	Y1245S	MUT	MPN	[54]
TET2	L1248P	MUT	AML	[11, 53]
TET2	G1256A	MUT	AML	[11]
TET2	G1256D	MUT	AML	[11]
TET2	G1256R	MUT	AML	[71]
TET2	R1261C	MUT	MPN, MDS/MPN, AML	[11, 53, 54, 60, 68, 75]
TET2	R1261H	MUT	MDS/MPN, AML	[54, 55, 76]

TET2	C1263R	MUT	AML	[11, 60]
TET2	N1266Y	MUT	AML	[11]
TET2	T1270A	MUT	AML	[11, 61]
TET2	C1271S	MUT	AML	[11, 53]
TET2	C1271W	MUT	MDS, MDS/MPN, AML	[11, 51, 77-79]
TET2	C1271Y	MUT	AML	[11, 53]
TET2	G1275E	MUT	MDS, MPN, AML	[11, 56, 62, 67]
TET2	G1275R	MUT	MPN	[11, 57]
TET2	G1282D	MUT	MDS, AML	[11, 51, 56]
TET2	S1286P	MUT	MDS	[66]
TET2	F1287L	MUT	MDS/MPN	[58]
TET2	G1288S	MUT	AML	[11, 62]
TET2	G1288V	MUT	MPN, MDS/MPN	[54, 56]
TET2	C1289Y	MUT	AML	[11, 53, 60]
TET2	W1291R	MUT	MDS, AML	[11, 50, 62]
TET2	S1292R	MUT	AML	[11, 51]
TET2	C1298W	MUT	AML	[5, 59]
TET2	C1298Y	MUT	MDS/MPN, AML	[11, 60]
TET2	K1299N	MUT	MDS/MPN	[58]
TET2	R1302G	MUT	MPN, AML	[58, 73, 80]
TET2	E1318G	MUT	MDS/MPN	[58]
TET2	L1340R	MUT	AML	[11, 53]
TET2	A1355P	MUT	MDS	[56]
TET2	R1359C	MUT	MPN, MDS/MPN, AML	[11, 54, 62, 71, 72, 81, 82]
TET2	R1359H	MUT	MDS/MPN, AML	[11, 62, 65]
TET2	L1360Q	MUT	MDS	[66]
TET2	G1361D	MUT	MDS/MPN, AML	[11, 60, 68]
TET2	G1361R	MUT	MPN	[54]
TET2	R1366H	MUT	AML	[60, 62, 66, 75, 83]
TET2	P1367S	MUT	MDS/MPN	[58]
TET2	F1368L	MUT	AML	[11, 60]
TET2	F1368Y	MUT	AML	[11, 51]
TET2	G1370V	MUT	AML	[11, 51]
TET2	T1372I	MUT	MPN	[11, 67]
TET2	D1376E	MUT	AML	[11, 60]
TET2	D1376G	MUT	AML	[66]
TET2	D1376N	MUT	MPN	[11, 57]
TET2	H1380Y	MUT	MDS, MDS/MPN, AML	[11, 53, 56, 60-62, 76]
TET2	H1382Y	MUT	AML	[11, 60]
TET2	D1384H	MUT	MDS, AML	[66, 69]
TET2	L1398R	MUT	MDS	[11, 50]
TET2	V1417F	MUT	MDS, MDS/MPN, AML, Lymphoid cancer	[6, 11, 41, 54, 60, 84]
TET2	P1419R	MUT	MDS, AML, Lymphoid cancer	[11, 53, 66, 84]
TET2	D1427Y	MUT	AML	[11, 62]

TET2	R1440Q	SNP		[57]
TET2	A1505T	SNP		[60]
TET2	A1505V	MUT	AML	[11]
TET2	E1513G	SNP		[62]
TET2	V1521L	SNP		[43]
TET2	S1556Y	MUT	AML	[59]
TET2	Y1579S	MUT	AML	[11, 62]
TET2	N1641I	MUT	AML	[11, 71]
TET2	G1697R	SNP		[60]
TET2	M1701I	SNP		[2, 9, 50, 53, 55, 56]
TET2	G1719R	MUT	MPN	[59]
TET2	L1721W	SNP		[2, 9, 50-53, 55]
TET2	P1723S	SNP		[9, 50, 52-55, 57]
TET2	H1757D	MUT	MDS/MPN	[54]
TET2	I1762V	SNP		[2, 9, 50-53, 55]
TET2	H1778R	SNP		[2, 9, 50-53, 55]
TET2	L1801F	MUT	AML	[11, 62]
TET2	C1811R	MUT	MDS/MPN	[54]
TET2	L1816F	SNP		[2]
TET2	H1817N	MUT	MPN, AML	[75, 85]
TET2	Q1828L	MUT	MPN	[54]
TET2	A1831S	SNP		[9]
TET2	V1833I	SNP		[60]
TET2	A1863D	MUT	AML	[11]
TET2	A1865G	MUT	AML	[62]
TET2	H1868Y	MUT	MPN, AML	[11]
TET2	S1870P	MUT	AML	[11, 53]
TET2	I1871S	MUT	AML	[11, 62]
TET2	I1873N	MUT	MDS	[56]
TET2	I1873T	MUT	MDS, MPN, MDS/MPN, AML	[11, 50, 53, 54, 58, 60, 62, 65, 68, 72, 80, 83, 85-88]
TET2	C1875R	MUT	MDS	[11, 50]
TET2	A1876V	MUT	AML	[11, 60]
TET2	H1881R	MUT	MPN, MDS/MPN	[54, 68, 81]
TET2	A1882P	MUT	AML	[11, 60]
TET2	T1884A	MUT	MDS/MPN, AML	[53, 65, 68, 76]
TET2	P1889L	MUT	AML	[11]
TET2	R1896M	MUT	AML	[11, 75, 80]
TET2	R1896S	MUT	MDS	[11, 50]
TET2	T1898F	MUT	MPN, MDS/MPN, AML, Lymphoid cancer	[11, 69, 80, 84, 89]
TET2	L1899P	MUT	AML	[11, 60]
TET2	V1900A	MUT	AML	[54, 76]
TET2	H1904R	MUT	MDS, MPN, AML	[53, 56, 85, 86]
TET2	G1913D	MUT	MDS	[11, 50, 58]
TET2	A1919V	MUT	MPN	[54]

TET2	R1926H	MUT	MDS/MPN	[54]
TET2	E1929K	SNP		[2]
TET2	P1941S	MUT	MDS/MPN, AML	[54, 56]
TET2	R1966H	MUT	MDS/MPN	[54]
TET2	R1974M	MUT	MDS/MPN	[54]
TET2	T1979P	MUT	MDS	[6]
TET2	R2000K	MUT	AML	[11, 54, 71]

Supplemental references

1. O. Abdel-Wahab, A. Pardanani, J. Patel, et al., "Concomitant analysis of EZH2 and ASXL1 mutations in myelofibrosis, chronic myelomonocytic leukemia and blast-phase myeloproliferative neoplasms," *Leukemia*, vol. 25, no. 7, pp. 1200-1202, 2011.
2. S. T. Sherry, M. H. Ward, M. Kholodov, et al., "dbSNP: the NCBI database of genetic variation," *Nucleic Acids Research*, vol. 29, no. 1, pp. 308-311, 2001.
3. R. Bejar, K. Stevenson, O. Abdel-Wahab, et al., "Clinical effect of point mutations in myelodysplastic syndromes," *New England Journal of Medicine*, vol. 364, no. 26, pp. 2496-2506, 2011.
4. S. Schnittger, C. Eder, S. Jeromin, et al., "ASXL1 exon 12 mutations are frequent in AML with intermediate risk karyotype and are independently associated with an adverse outcome," *Leukemia*, vol. 27, no. 1, pp. 82-91, 2013.
5. A. M. Jankowska, H. Makishima, R. V. Tiu, et al., "Mutational spectrum analysis of chronic myelomonocytic leukemia includes genes associated with epigenetic regulation: UTX, EZH2, and DNMT3A," *Blood*, vol. 118, no. 14, pp. 3932-3941, 2011.
6. Y. Sugimoto, M. A. Sekeres, H. Makishima, et al., "Cytogenetic and molecular predictors of response in patients with myeloid malignancies without del[5q] treated with lenalidomide," *Journal of Hematology and Oncology*, vol. 5, p. 4, 2012.
7. Y. Sugimoto, H. Muramatsu, H. Makishima, et al., "Spectrum of molecular defects in juvenile myelomonocytic leukaemia includes ASXL1 mutations," *British Journal of Haematology*, vol. 150, no. 1, pp. 83-87, 2010.
8. M. Brecqueville, J. Rey, F. Bertucci, et al., "Mutation analysis of ASXL1, CBL, DNMT3A, IDH1, IDH2, JAK2, MPL, NF1, SF3B1, SUZ12, and TET2 in myeloproliferative neoplasms," *Genes Chromosomes and Cancer*, vol. 51, no. 8, pp. 743-755, 2012.
9. L. Martínez-Avilés, C. Besses, A. Álvarez-Larrán, E. Torres, S. Serrano, and B. Bellosillo, "TET2, ASXL1, IDH1, IDH2, and c-CBL genes in JAK2- and MPL-negative myeloproliferative neoplasms," *Annals of Hematology*, vol. 91, no. 4, pp. 533-541, 2012.
10. J. Boultwood, J. Perry, R. Zaman, et al., "High-density single nucleotide polymorphism array analysis and ASXL1 gene mutation screening in chronic myeloid leukemia during disease progression," *Leukemia*, vol. 24, no. 6, pp. 1139-1145, 2010.

11. S. A. Forbes, N. Bindal, S. Bamford, et al., "COSMIC: mining complete cancer genomes in the Catalogue of Somatic Mutations in Cancer," *Nucleic Acids Research*, vol. 39, no. suppl 1, pp. D945-D950, 2011.

12. B. Pérez, O. Kosmider, B. Cassinat, et al., "Genetic typing of CBL, ASXL1, RUNX1, TET2 and JAK2 in juvenile myelomonocytic leukaemia reveals a genetic profile distinct from chronic myelomonocytic leukaemia," *British Journal of Haematology*, vol. 151, no. 5, pp. 460-468, 2010.

13. P. Guglielmelli, F. Biamonte, J. Score, et al., "EZH2 mutational status predicts poor survival in myelofibrosis," *Blood*, vol. 118, no. 19, pp. 5227-5234, 2011.

14. T. Ernst, A. J. Chase, J. Score, et al., "Inactivating mutations of the histone methyltransferase gene EZH2 in myeloid disorders," *Nature Genetics*, vol. 42, no. 8, pp. 722-726, 2010.

15. G. Nikoloski, S. M. Langemeijer, R. P. Kuiper, et al., "Somatic mutations of the histone methyltransferase gene EZH2 in myelodysplastic syndromes," *Nature Genetics*, vol. 42, no. 8, pp. 665-667, 2010.

16. V. Grossmann, U. Bacher, A. Kohlmann, et al., "EZH2 mutations and their association with PICALM-MLLT10 positive acute leukaemia," *British Journal of Haematology*, vol. 157, no. 3, pp. 387-390, 2012.

17. R. D. Morin, N. A. Johnson, T. M. Severson, et al., "Somatic mutations altering EZH2 (Tyr641) in follicular and diffuse large B-cell lymphomas of germinal-center origin," *Nature Genetics*, vol. 42, no. 2, pp. 181-185, 2010.

18. D. Capello, A. Gloghini, M. Martini, et al., "Mutations of CD79A, CD79B and EZH2 genes in immunodeficiency-related non-Hodgkin lymphomas," *British Journal of Haematology*, vol. 152, no. 6, pp. 777-780, 2011.

19. M. T. McCabe, A. P. Graves, G. Ganji, et al., "Mutation of A677 in histone methyltransferase EZH2 in human B-cell lymphoma promotes hypertrimethylation of histone H3 on lysine 27 (H3K27)," *Proceedings of the National Academy of Sciences of the United States*, vol. 109, no. 8, pp. 2989-2994, 2012.

20. J. Wang, X. Ai, R. P. Gale, et al., "TET2, ASXL1 and EZH2 mutations in Chinese with myelodysplastic syndromes," *Leukemia Research*, vol. 37, no. 3, pp. 305-311, 2013.

21. V. Grossmann, A. Kohlmann, C. Eder, et al., “Molecular profiling of chronic myelomonocytic leukemia reveals diverse mutations in >80% of patients with TET2 and EZH2 being of high prognostic relevance,” *Leukemia*, vol. 25, no. 5, pp. 877-879, 2011.

22. H. Makishima, A. M. Jankowska, R. V. Tiu, et al., “Novel homo- and hemizygous mutations in EZH2 in myeloid malignancies,” *Leukemia*, vol. 24, no. 10, pp. 1799-1804, 2010.

23. I. Fried, C. Bodner, M. M. Pichler, et al., “Frequency, onset and clinical impact of somatic DNMT3A mutations in therapy-related and secondary acute myeloid leukemia,” *Haematologica*, vol. 97, no. 2, pp. 246-250, 2012.

24. T. J. Ley, L. Ding, M. J. Walter, et al., “DNMT3A mutations in acute myeloid leukemia,” *New England Journal of Medicine*, vol. 363, no. 25, pp. 2424-2433, 2010.

25. X. J. Yan, J. Xu, Z. H. Gu, et al., “Exome sequencing identifies somatic mutations of DNA methyltransferase gene DNMT3A in acute monocytic leukemia,” *Nature Genetics*, vol. 43, no. 4, pp. 309-315, 2011.

26. Y. Shen, Y. M. Zhu, X. Fan, et al., “Gene mutation patterns and their prognostic impact in a cohort of 1185 patients with acute myeloid leukemia,” *Blood*, vol. 118, no. 20, pp. 5593-5603, 2011.

27. J. Marková, P. Michková, K Burčková, et al., “Prognostic impact of DNMT3A mutations in patients with intermediate cytogenetic risk profile acute myeloid leukemia,” *European Journal of Haematology*, vol. 88, no. 2, pp. 128-135, 2012.

28. V, Grossmann, C. Haferlach, S. Weissmann, et al., “The molecular profile of adult T-cell acute lymphoblastic leukemia: mutations in RUNX1 and DNMT3A are associated with poor prognosis in T-ALL,” *Genes Chromosomes and Cancer*, vol. 52, no. 4, pp. 410-422, 2013.

29. M. J. Walter, L. Ding, D. Shen, et al., “Recurrent DNMT3A mutations in patients with myelodysplastic syndromes,” *Leukemia*, vol. 25, no. 7, 1153-1158, 2011.

30. F. Thol, F. Damm, A. Lüdeking, et al., “Incidence and prognostic influence of DNMT3A mutations in acute myeloid leukemia,” *Journal of Clinical Oncology*, vol. 29, no. 21, pp. 2889-2896, 2011.

31. M. Brecqueville, N. Cervera, V. Gelsi-Boyer, et al., “Rare mutations in DNMT3A in myeloproliferative neoplasms and myelodysplastic syndromes,” *Blood Cancer Journal*, vol. 1, no. 5, p. e18, 2011.

32. N. Rao, C. M. Butcher, I. D. Lewis, et al., "Clonal and lineage analysis of somatic DNMT3A and JAK2 mutations in a chronic phase polycythemia vera patient," *British Journal of Haematology*, vol. 156, no. 2, pp. 268-270, 2012.

33. Y. Yamashita, J. Yuan, I. Suetake, et al., "Array-based genomic resequencing of human leukemia," *Oncogene*, vol. 29, no. 25, pp. 3723-3731, 2010.

34. F. Stegelmann, L. Bullinger, R. F. Schlenk, et al., "DNMT3A mutations in myeloproliferative neoplasms," *Leukemia*, vol. 25, no. 7, pp. 1217-1219, 2011.

35. F. Thol, M. Heuser, F. Damm, J. H. Klusmann, K. Reinhardt, and D. Reinhardt, "DNMT3A mutations are rare in childhood acute myeloid leukemia," *Haematologica*, vol. 96, no. 8, pp. 1238-1240, 2011.

36. J. Lin, D. M. Yao, J. Qian, et al., "Recurrent DNMT3A R882 mutations in Chinese patients with acute myeloid leukemia and myelodysplastic syndrome," *PLoS One*, vol. 6, no. 10, p. e26906, 2011.

37. O. LaRochelle, S. Bertoli, F. Vergez, et al., "Do AML patients with DNMT3A exon 23 mutations benefit from idarubicin as compared to daunorubicin? A single center experience," *Oncotarget*, vol. 2, no. 11, 850-861, 2011.

38. Y. Li, D. F. Zhang, S. W. Zhang, Y. Zeng, and Y. G. Yao, "Screening for mutation R882 in the DNMT3A gene in Chinese patients with hematological disease," *International Journal of Hematology*, vol. 96, no. 2, pp. 229-233, 2012.

39. Q. Lu, Y. Chen, H. Wang, and Z. Li, "DNMT3A mutations and clinical features in Chinese patients with acute myeloid leukemia," *Cancer Cell International*, vol. 13, no. 1, p. 1, 2013.

40. M. Ewalt, N. G. Galili, M. Mumtaz, et al., "DNMT3a mutations in high-risk myelodysplastic syndrome parallel those found in acute myeloid leukemia," *Blood Cancer Journal*, vol. 1, no. 3, p. e9, 2011.

41. M. A. Sekeres, R. V. Tiu, R. Komrokji, et al., "Phase 2 study of the lenalidomide and azacitidine combination in patients with higher-risk myelodysplastic syndromes," *Blood*, vol. 120, no. 25, pp. 4945-4951, 2012.

42. F. Thol, C. Winschel, A. Lüdeking, et al., "Rare occurrence of DNMT3A mutations in myelodysplastic syndromes," *Haematologica*, vol. 96, no. 12, pp. 1870-1873, 2011.

43. T. Ottone, L. Cicconi, S. K. Hasan, et al., "Comparative molecular analysis of therapy-related and de novo acute promyelocytic leukemia," *Leukemia Research*, vol. 36, no. 4, pp. 474-478, 2012.

44. A. Pezzi, L. Moraes, V. Valim, et al., "DNMT3A Mutations in Patients with Acute Myeloid Leukemia in South Brazil," *Advances in Hematology*, vol. 2012, Article ID 697691, 8 pages, 2012.

45. A. H. Shih, S. S. Chung, E. K. Dolezal EK, et al., "Mutational analysis of therapy-related myelodysplastic syndromes and acute myelogenous leukemia," *Haematologica*, vol. 98, no. 6, pp. 908-912, 2013.

46. O. Abdel-Wahab, A. Pardanani, R. Rampal, T. L. Lasho, R. L. Levine, and A. Tefferi, "DNMT3A mutational analysis in primary myelofibrosis, chronic myelomonocytic leukemia and advanced phases of myeloproliferative neoplasms," *Leukemia*, vol. 25, no. 7, pp. 1219-1220, 2011.

47. M. W. Wlodarski, J. Mötter, T. A. Gorr, et al., "Abnormal promoter DNA methylation in juvenile myelomonocytic leukemia is not caused by mutation in DNMT3A," *Blood*, vol. 118, no. 16, pp. 4490-4491, 2011.

48. L. Couronné, C. Bastard, and O. A. Bernard, "TET2 and DNMT3A mutations in human T-cell lymphoma," *New England Journal of Medicine*, vol. 366, no. 1, pp. 95-96, 2012.

49. C. Simon, J. Chagraoui, J. Krosl, et al., "A key role for EZH2 and associated genes in mouse and human adult T-cell acute leukemia," *Genes and Development*, vol. 26, no. 7, pp. 651-656, 2012.

50. S. M. Langemeijer, R. P. Kuiper, M. Berends, et al., "Acquired mutations in TET2 are common in myelodysplastic syndromes," *Nature Genetics*, vol. 41, no. 7, pp. 838-842, 2009.

51. O. Nibourel, O. Kosmider, M. Cheok, et al., "Incidence and prognostic value of TET2 alterations in de novo acute myeloid leukemia achieving complete remission," *Blood*, vol. 116, no. 7, pp. 1132-1135, 2010.

52. S. M. Langemeijer, J. H. Jansen, J. Hooijer, et al., "TET2 mutations in childhood leukemia," *Leukemia*, vol. 25, no. 1, pp. 189-192, 2011.

53. S. Weissmann, T. Alpermann, V. Grossmann, et al., "Landscape of TET2 mutations in acute myeloid leukemia," *Leukemia*, vol. 26, no. 5, pp. 934-942, 2012.

54. O. Abdel-Wahab, A. Mullally, C Hedvat, et al., "Genetic characterization of TET1, TET2, and TET3 alterations in myeloid malignancies," *Blood*, vol. 114, no. 1, pp. 144-147, 2009.

55. A. Kohlmann, H. U. Klein, S. Weissmann, et al., "The Interlaboratory RObustness of Next-generation sequencing (IRON) study: a deep sequencing investigation of TET2, CBL and KRAS mutations by an international consortium involving 10 laboratories," *Leukemia*, vol. 25, no. 12, pp. 1840-1848, 2011.

56. A. E. Smith, A. M. Mohamedali, A. Kulasekararaj, et al., "Next-generation sequencing of the TET2 gene in 355 MDS and CMML patients reveals low-abundance mutant clones with early origins, but indicates no definite prognostic value," *Blood*, vol. 116, no. 19, pp. 3923-3932, 2010.

57. D. Olcaydu, E. Rumi, A. Harutyunyan, et al., "The role of the JAK2 GGCC haplotype and the TET2 gene in familial myeloproliferative neoplasms," *Haematologica*, vol. 96, no. 3, pp. 367-374, 2011.

58. A. M. Jankowska, H. Szpurka, R. V. Tiu, et al., "Loss of heterozygosity 4q24 and TET2 mutations associated with myelodysplastic/myeloproliferative neoplasms," *Blood*, vol. 113, no. 25, pp. 6403-6410, 2009.

59. H. Makishima, A. M. Jankowska, M. A. McDevitt, et al., "CBL, CBLB, TET2, ASXL1, and IDH1/2 mutations and additional chromosomal aberrations constitute molecular events in chronic myelogenous leukemia," *Blood*, vol. 117, no. 21, pp. e198-e206, 2011.

60. K. H. Metzeler, K. Maharry, M. D. Radmacher, et al., "TET2 mutations improve the new European LeukemiaNet risk classification of acute myeloid leukemia: a Cancer and Leukemia Group B study," *Journal of Clinical Oncology*, vol. 29, no. 10, pp. 1373-1381, 2011.

61. J. Nomdedéu, M. Hoyos, M. Carricondo, et al., "Adverse impact of IDH1 and IDH2 mutations in primary AML: experience of the Spanish CETLAM group," *Leukemia Research*, vol. 36, no. 8, pp. 990-997, 2012.

62. W. C. Chou, S. C. Chou, C. Y. Liu, et al., "TET2 mutation is an unfavorable prognostic factor in acute myeloid leukemia patients with intermediate-risk cytogenetics," *Blood*, vol. 118, no. 14, pp. 3803-3810, 2011.

63. A. Thiel, M. Beier, D, Ingenhag, et al., “Comprehensive array CGH of normal karyotype myelodysplastic syndromes reveals hidden recurrent and individual genomic copy number alterations with prognostic relevance,” *Leukemia*, vol. 25, no. 3, pp. 387-399, 2011.

64. C. Pérez, N. Martínez-Calle, J. I. Martín-Subero, et al., “TET2 mutations are associated with specific 5-methylcytosine and 5-hydroxymethylcytosine profiles in patients with chronic myelomonocytic leukemia,” *PLoS One*, vol. 7, no. 2, p. e31605, 2012.

65. O. Kosmider, V. Gelsi-Boyer, M. Ciudad, et al., “TET2 gene mutation is a frequent and adverse event in chronic myelomonocytic leukemia,” *Haematologica*, vol. 94, no. 12, pp. 1676-1681, 2009.

66. J. Rocquain, N. Carbuccia, V. Trouplin, et al., “Combined mutations of ASXL1, CBL, FLT3, IDH1, IDH2, JAK2, KRAS, NPM1, NRAS, RUNX1, TET2 and WT1 genes in myelodysplastic syndromes and acute myeloid leukemias,” *BMC Cancer*, vol. 10, p. 401, 2010.

67. F. X. Schaub, R. Looser, S. Li, et al., “Clonal analysis of TET2 and JAK2 mutations suggests that TET2 can be a late event in the progression of myeloproliferative neoplasms,” *Blood*, vol. 115, no. 10, pp. 2003-2007, 2010.

68. A. Kohlmann, V. Grossmann, H. U. Klein, et al., “Next-generation sequencing technology reveals a characteristic pattern of molecular mutations in 72.8% of chronic myelomonocytic leukemia by detecting frequent alterations in TET2, CBL, RAS, and RUNX1,” *Journal of Clinical Oncology*, vol. 28, no. 24, pp. 3858-3865, 2010.

69. O. Kosmider, E. Delabesse, V. M. de Mas, et al., “TET2 mutations in secondary acute myeloid leukemias: a French retrospective study,” *Haematologica*, vol. 96, no. 7, pp. 1059-1063, 2011.

70. J. Flach, F. Dicker, S. Schnittger, A. Kohlmann, T. Haferlach, and C. Haferlach, “Mutations of JAK2 and TET2, but not CBL are detectable in a high portion of patients with refractory anemia with ring sideroblasts and thrombocytosis,” *Haematologica*, vol. 95, no. 3, pp. 518-519, 2010.

71. O. Abdel-Wahab, T. Manshouri, J. Patel, et al., “Genetic analysis of transforming events that convert chronic myeloproliferative neoplasms to leukemias,” *Cancer Research*, vol. 70, no. 2, pp. 447-452, 2010.

72. A. Tefferi, A. Pardanani, K. H. Lim, et al., “TET2 mutations and their clinical correlates in polycythemia vera, essential thrombocythemia and myelofibrosis,” *Leukemia*, vol. 23, no. 5, pp. 905-911, 2009.

73. E. Pronier, C. Almire, H. Mokrani, et al., “Inhibition of TET2-mediated conversion of 5-methylcytosine to 5-hydroxymethylcytosine disturbs erythroid and granulomonocytic differentiation of human hematopoietic progenitors,” *Blood*, vol. 118, no. 9, pp. 2551-2555, 2011.

74. P. A. Beer, F. Delhommeau, J. P. LeCouédic, et al., “Two routes to leukemic transformation after a JAK2 mutation-positive myeloproliferative neoplasm,” *Blood*, vol. 115, no. 14, pp. 2891-2900, 2010.

75. K. Moran-Crusio, L. Reavie, A. Shih, et al., “Tet2 loss leads to increased hematopoietic stem cell self-renewal and myeloid transformation,” *Cancer Cell*, vol. 20, no. 1, pp. 11-24, 2011.

76. V. I. Gaidzik, P. Paschka, D. Späth, et al., “TET2 mutations in acute myeloid leukemia (AML): results from a comprehensive genetic and clinical analysis of the AML study group,” *Journal of Clinical Oncology*, vol. 30, no. 12, pp. 1350-1357, 2012.

77. M. T. Voso, E. Fabiani, A. Piciocchi, et al., “Role of BCL2L10 methylation and TET2 mutations in higher risk myelodysplastic syndromes treated with 5-azacytidine,” *Leukemia*, vol. 25, no. 12, pp. 1910-1913, 2011.

78. K. Yoshida, M. Sanada, Y. Shiraishi, et al., “Frequent pathway mutations of splicing machinery in myelodysplasia,” *Nature*, vol. 478, no. 7367, pp. 64-69, 2011.

79. U. Bacher, S. Weissmann, A. Kohlmann, et al., “TET2 deletions are a recurrent but rare phenomenon in myeloid malignancies and are frequently accompanied by TET2 mutations on the remaining allele,” *British Journal of Haematology*, vol. 156, no. 1, pp. 67-75, 2012.

80. F. Delhommeau, S. Dupont, V. Della Valle, et al., “Mutation in TET2 in myeloid cancers,” *New England Journal of Medicine*, vol. 360, no. 22, pp. 2289-2301, 2009.

81. A. Tefferi, R. L. Levine, K. H. Lim, et al., “Frequent TET2 mutations in systemic mastocytosis: clinical, KITD816V and FIP1L1-PDGFRA correlates,” *Leukemia*, vol. 23, no. 5, pp. 900-904, 2009.

82. V. Gelsi-Boyer, V. Trouplin, J. Roquain, et al., “ASXL1 mutation is associated with poor prognosis and acute transformation in chronic myelomonocytic leukaemia,” *British Journal of Haematology*, vol. 151, no. 4, 365-375, 2010.

83. L. Couronné, E. Lippert, J. Andrieux, et al., "Analyses of TET2 mutations in post-myeloproliferative neoplasm acute myeloid leukemias," *Leukemia*, vol. 24, no. 1, pp. 201-203, 2010.

84. C. Quivoron, L. Couronné, V. Della Valle, et al., "TET2 inactivation results in pleiotropic hematopoietic abnormalities in mouse and is a recurrent event during human lymphomagenesis," *Cancer Cell*, vol. 20, no. 1, pp. 25-38, 2011.

85. M. Ko, Y. Huang, A. M. Jankowska, et al., "Impaired hydroxylation of 5-methylcytosine in myeloid cancers with mutant TET2," *Nature*, vol. 468, no. 7325, pp. 839-843, 2010.

86. F. Traina, V. Visconte, A. M. Jankowska, et al., "Single nucleotide polymorphism array lesions, TET2, DNMT3A, ASXL1 and CBL mutations are present in systemic mastocytosis," *PLoS One*, vol. 7, no. 8, p. e43090, 2012.

87. N. Konstandin, S. Bultmann, A. Szwagierczak, et al., "Genomic 5-hydroxymethylcytosine levels correlate with TET2 mutations and a distinct global gene expression pattern in secondary acute myeloid leukemia," *Leukemia*, vol. 25, no. 10, pp. 1649-1652, 2011.

88. O. Kosmider, V. Gelsi-Boyer, M. Cheok, et al., "TET2 mutation is an independent favorable prognostic factor in myelodysplastic syndromes (MDSs)," *Blood*, vol. 114, no. 15, pp. 3285-3291, 2009.

89. C. Pérez, N. Martínez-Calle, J. I. Martín-Subero, et al., "TET2 mutations are associated with specific 5-methylcytosine and 5-hydroxymethylcytosine profiles in patients with chronic myelomonocytic leukemia," *PLoS One*, vol. 7, no. 2, p. e31605, 2012.
